# Supplementary figures and images for: Host cell-specific metabolism of linoleic acid controls Toxoplasma gondii growth in cell culture
Source: bioRxiv. 2024 Mar 25:2024.03.22.586332. Preprint. [Version 2] doi: 10.1101/2024.03.22.586332 (PMC10983968; doi:10.1101/2024.03.22.586332)

**A**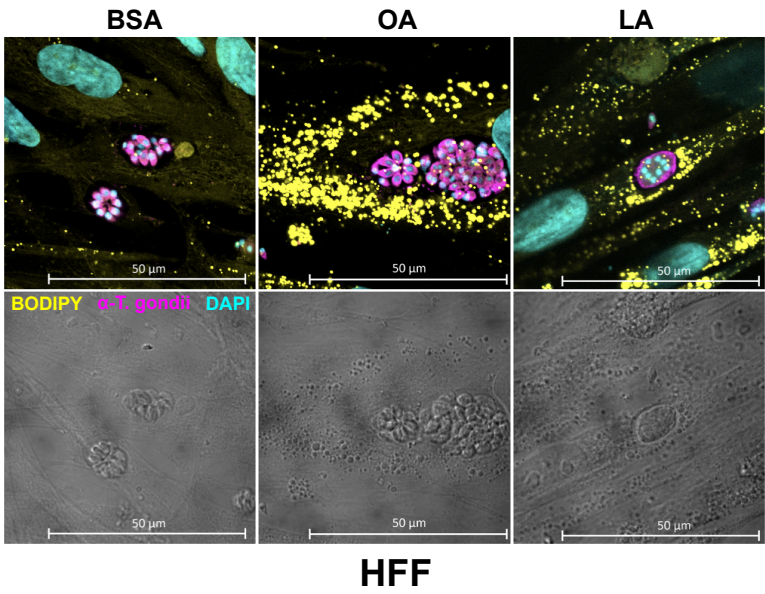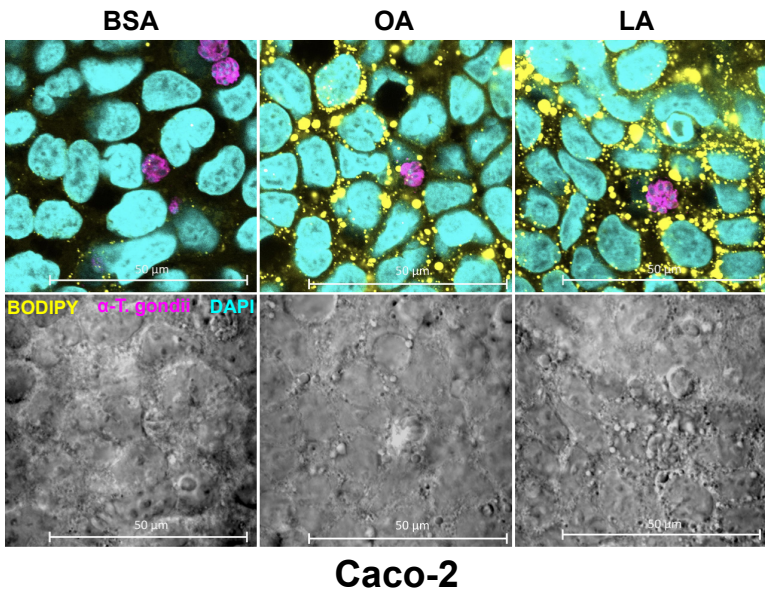**C**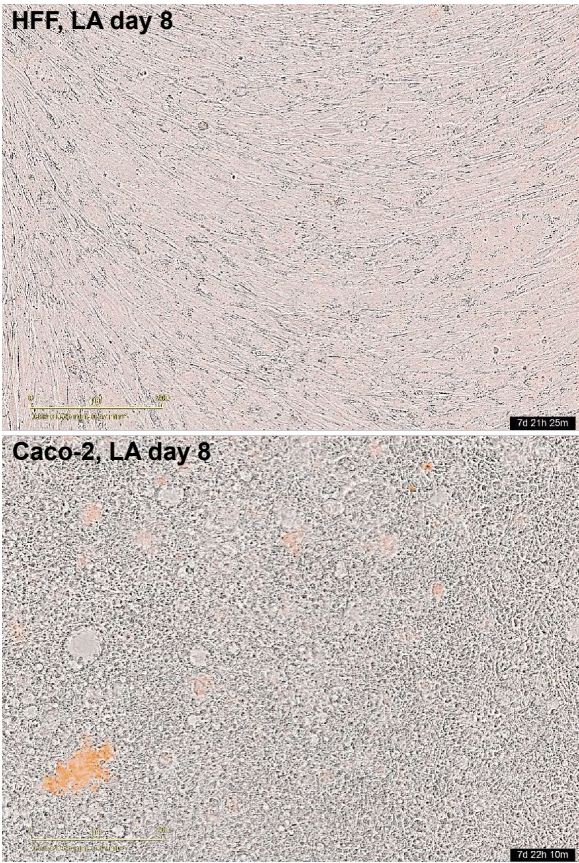**B**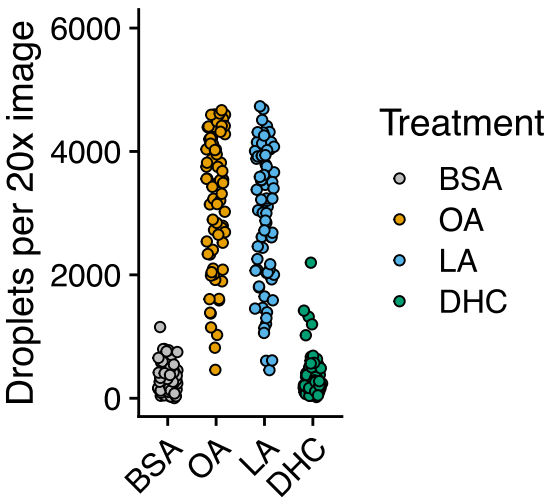

Supplement: Supplement 1 — Figure S3. Related to Figure 3: In HFFs, linoleic acid disrupts parasite membranes and increases lipid droplets. (a) 100x confocal fluorescence microscopy images of host lipid droplets in ME49 Δhpt luciferase-infected HFFs and Caco-2s following 24 hours of treatment with 350 μM OA, 350 μM LA, or an equal volume of BSA (final concentration 75 μM). Magenta = T. gondii, yellow = lipid droplets, cyan = DNA. (b) Incucyte-based quantification of lipid droplets in uninfected Caco-2s after 24 hours of treatment with 350 μM OA, 350 μM LA, 500 nM DHC, or an equal volume of BSA (final concentration 75 μM). (c) Incucyte images at 20x magnification of intact HFF and Caco-2 monolayers after 1 week of infection and fatty acid treatment. Red = mCherry-positive parasites. [file media-1.pdf]

**A**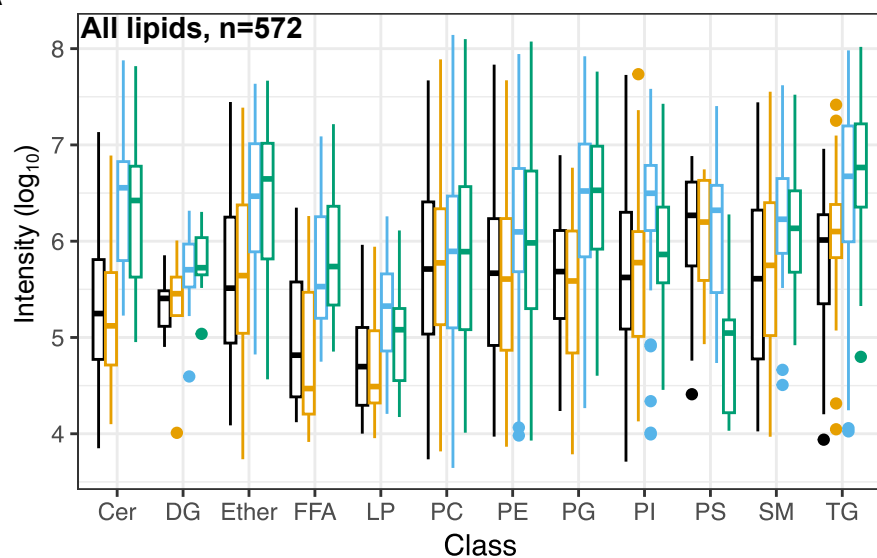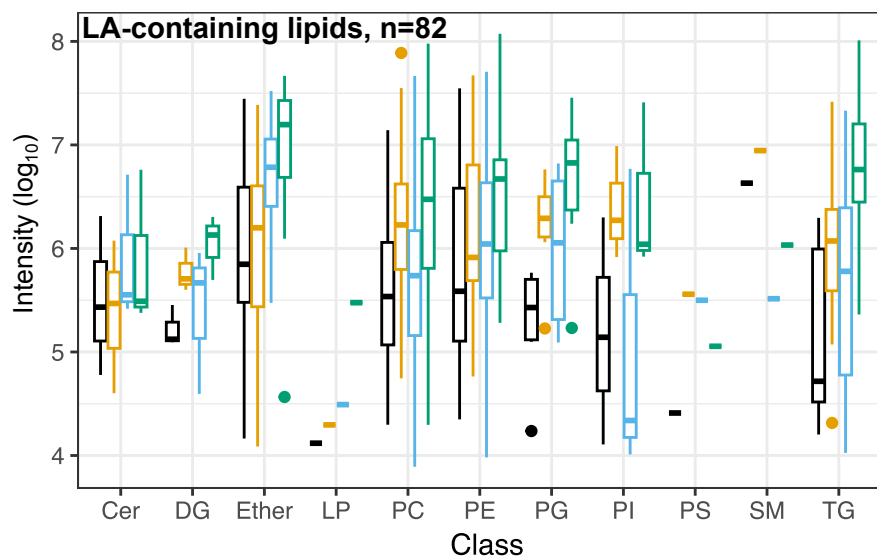

BSA HFF LA HFF BSA Caco-2 LA Caco-2

**B**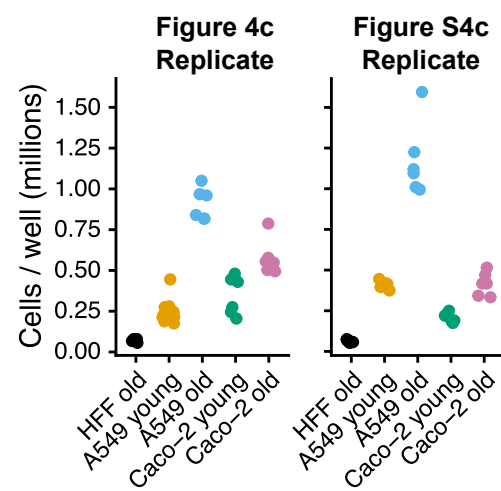**C**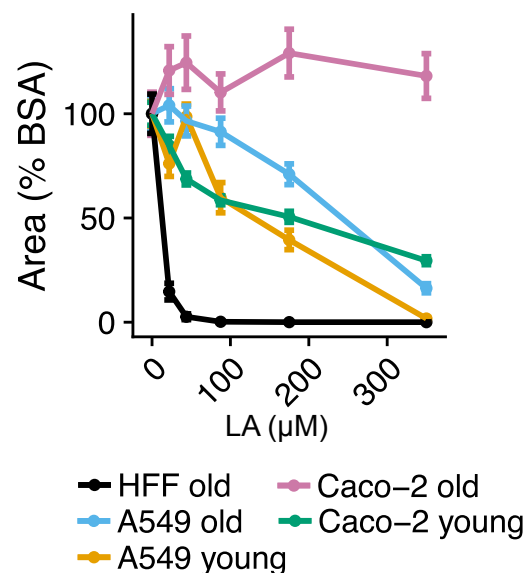**D**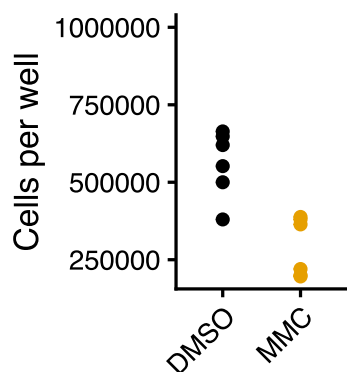**E**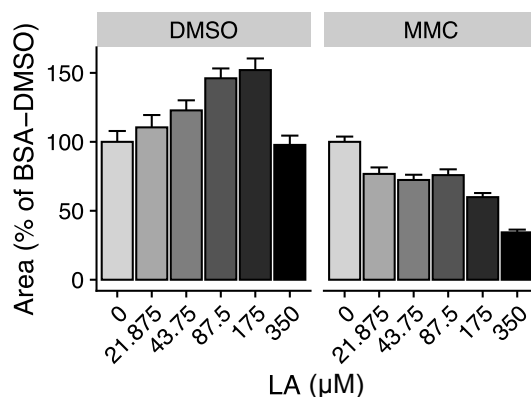

Supplement: Supplement 2 — Figure S4. Related to Figure 4: High linoleic acid metabolism in Caco-2 cells controls parasite load. (a) Log10-transformed abundances of lipids divided by lipid class. Cer = ceramides, DG = diacylglycerols, Ether = ether phospholipids, FFA = free fatty acids, LP = lysophospholipids, PC = phosphatidylcholine, PE = phosphatidylethanolamine, PG = phosphatidylglycerol, PI = phosphatidylinositol, PS = phosphatidylserine, SM = sphingomyelin, TG = triacylglycerol. All lipids (n=572) are included in the top plot. The bottom plot is restricted to LA-containing lipids (n=82). (b) Cells from the same 48-well plates used in Figure 4c were trypsinized and counted (n=6 per plate). (c) A second replicate experiment of the data shown in Fig. 4c, the cell types and abundance experiment. (d) Similar to (b), cell counts from the 48-well plates used in Fig. 4d, the mitomycin C experiment. (e) A second replicate experiment of the data shown in Fig. 4d, the mitomycin C experiment. [file media-2.pdf]

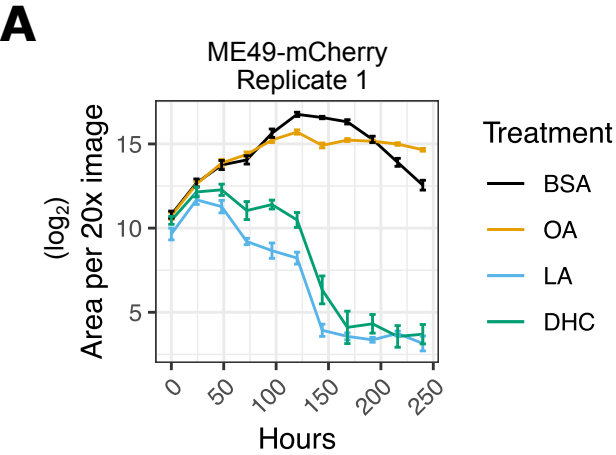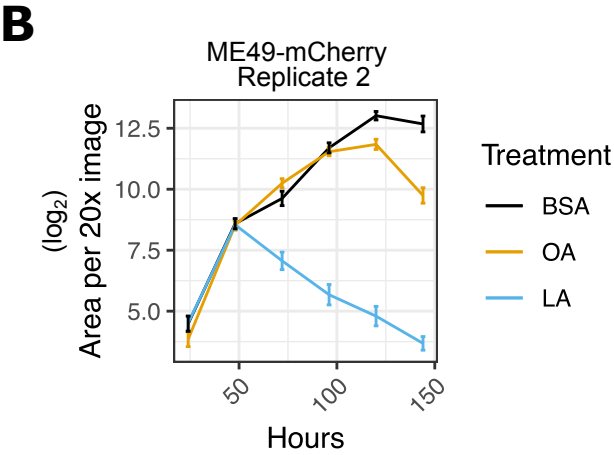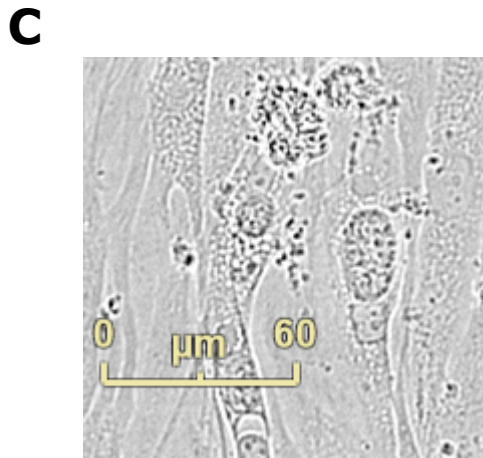

Supplement: Supplement 3 — Figure S2. Related to Figure 2: Linoleic acid is parasiticidal. (a, b) Additional experiments supporting conclusions from Figure 2a but with ME49-mCherry parasites. (c) A representative Incucyte phase channel image of intact HFF monolayer infected with EGS parasites after 1 week of growth at pH 8.0 in a humidified incubator without CO2. [file media-3.pdf]

**A**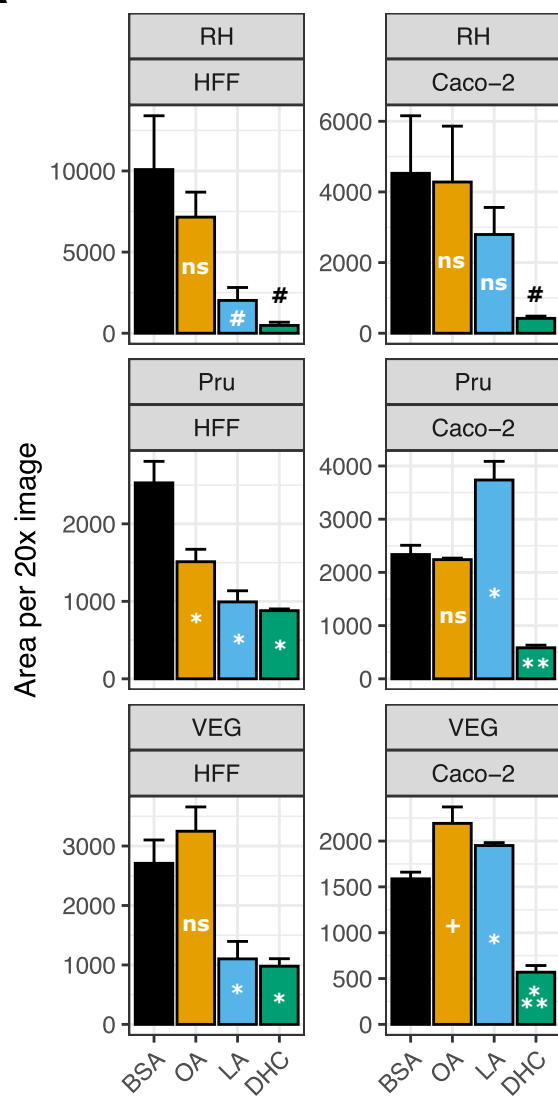**B**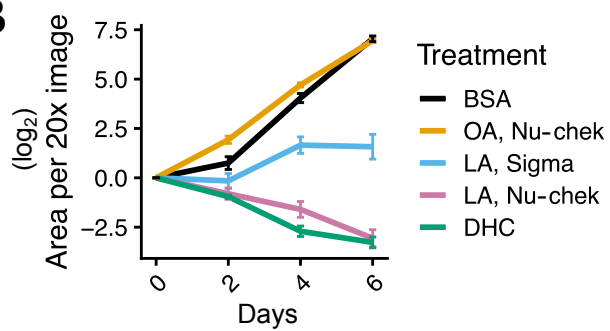**C**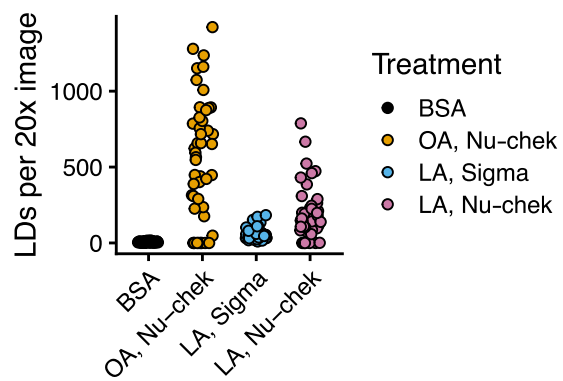**D**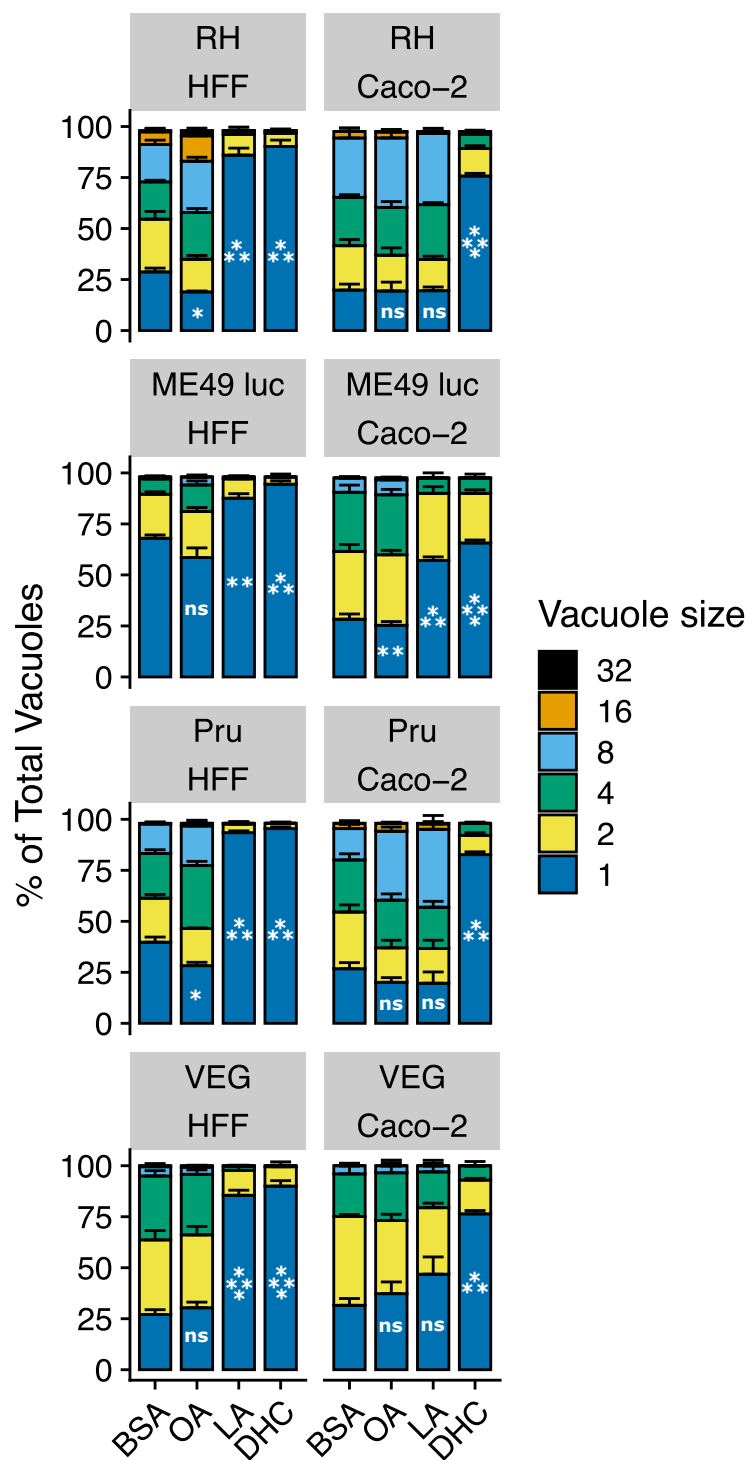

Supplement: Supplement 4 — Figure S1. Related to Figure 1: Linoleic acid slows parasite growth in HFF cells but not in Caco-2 cells. (a) A second replicate experiment of Fig. 1a. T. gondii abundance after 3 days of treatment with 350 μM OA, 350 μM LA, 500 nM DHC, or an equal volume of BSA (final concentration 75 μM) in confluent HFFs or Caco-2 cells. After fixing and staining parasites red with immunofluorescence, 16 technical replicate images for each of 3 biological replicate wells were collected at 20x magnification on the Incucyte imaging system. Red fluorescence area was calculated for each technical replicate and averaged to obtain a value for each biological replicate. The mean of biological replicates is displayed on the y-axis +/− SE. #, p < 0.15; +, p < 0.1; *, p < 0.05; **, p < 0.01; ***, p < 0.001 by Student’s t-test. (b) LA vendor influences LA potency. RH-mCherry growth was measured over time using the Incucyte, in the presence of the treatments listed in (a). (c) The Incucyte was used to count BODIPY 493/503-positive lipid droplets in HFFs in the presence of treatments listed in (a). (d) A second replicate experiment of Fig. 1b. T. gondii replication assessed by parasitophorous vacuole (PV) size after 24 hours of treatments noted in (a). Number of parasites per vacuole are shown as mean percentages of total PVs +/− SE. At least 100 vacuoles were counted for each of 3 biological replicate wells per condition. *, p < 0.05; **, p < 0.01; ***, p < 0.001; ****, p < 0.0001 by Student’s t-test for percent of single-parasite vacuoles. [file media-4.pdf]
