# Supplementary material for: Host cell-specific metabolism of linoleic acid controls Toxoplasma gondii growth in cell culture
Source: bioRxiv. 2024 Mar 25:2024.03.22.586332. Preprint. [Version 2] doi: 10.1101/2024.03.22.586332 (PMC10983968; doi:10.1101/2024.03.22.586332)
Supplement: Supplement 5 [file NIHPP2024.03.22.586332v2-supplement-5.pdf]

bioRxiv preprint doi: https://doi.org/10.1101/2024.03.22.586332; this version posted March 25, 2024. The copyright holder for this preprint (which was not certified by peer review) is the author/funder, who has granted bioRxiv a license to display the preprint in perpetuity. It is made available under aCC-BY 4.0 International license.

**Supplementary Information 1. Lipidomics data table and sample metadata.** Per-sample metabolite peak areas are reported for both positive and negative ion mode mass spectrometry runs. Sample metadata is included, as is a readme.
